# Emotional Intelligence in Carriers of Different СОМТ, BDNF, DRD2 and HTR2A Genotypes

**DOI:** 10.11621/pir.2022.0206

**Published:** 2022-06-30

**Authors:** Elena V. Vorobyeva, Ekaterina M. Kovsh, Vladimir V. Kosonogov

**Affiliations:** a Southern Federal University, Rostov-on-Don, Russia; b HSE University, Moscow, Russia

**Keywords:** Emotional intelligence, genetics, СОМТ, BDNF, DRD2, HTR2A

## Abstract

**Background:**

Emotional intelligence is the ability to quickly and correctly recognize the emotional expressions of other people and to express and manage one’s own emotions. It contributes to the success of a person in activities related to communication and interaction with people. Emotional intelligence has been studied largely in the context of organizational and education psychology, but less is known about the influence of genetics on it.

**Objective:**

We aim to study emotional intelligence in carriers of different СОМТ, BDNF, DRD2, and HTR2A genotypes.

**Design:**

We used three methods to measure emotional intelligence. Mayer-Salovey-Caruso Emotional Intelligence Test is a set of tasks with forced choice and frequency-based correct responses. We also applied two self-report questionnaires by Lyusin and Hall. We recruited 280 participants who took part in all three measures. We also identified their genotypes of the СОМТ, BDNF, DRD2, and HTR2A genes.

**Results:**

Carriers of the Val/Met genotype of the COMT gene, A/A genotype of the HTR2A gene and C/C genotype of the DRD2 gene showed the highest level of emotional intelligence, while no differences were found between carriers of the BDNF genotypes. These data were obtained by using the Mayer-Salovey-Caruso Emotional Intelligence Test. Self-report scores of emotional intelligence did not differ between carriers of different genotypes across all four of the genes in question.

**Conclusion:**

Mayer-Salovey-Caruso Emotional Intelligence Test scores were differed for carriers of some genotypes, whereas self-reported emotional intelligence scores did not differ between according to genotype.

## Introduction

Emotional intelligence is the ability to quickly and correctly recognize the emotional expressions of other people and to express and manage one’s own emotions. Emotional intelligence contributes to the success of a person in activities related to communication and interaction with people (Mayer et al., 2004; Lanthem, 2021). Emotional intelligence has been shown to correlate with a general factor of personality, which indicates a high content similarity of these constructs (Anglim et al., 2020). The level of emotional intelligence is an important factor in social effectiveness, decision-making, and prevention of various risks (Pavlova et al., 2013; Cha et al., 2013; Hosseini et al., 2013; van der Linden et al., 2017; Kundi et al., 2021). Emotional intelligence is considered a resource for human development and well-being. There is a difference in emotional intelligence between men and women. That is, emotional intelligence seems to be higher in females (Sergienko et al., 2020; Fernández et al., 2020).

The concept of emotional intelligence finds its origin in Thorndike’s theory of social intelligence. This concept involved the ability to understand, manage, and interact wisely with other people. In the latter part of the 20th century, within the framework of Gardner’s ideas about interpersonal intelligence, was formed the idea that emotional intelligence pertains to the ability of an individual to understand the intentions, motivations and desires of both himself and others (quoted by Smith et al., 2018). In modern studies, researchers attempt to study emotional intelligence, among other things, within the context of theory of mind by choosing certain categories of respondents for research (Ferguson et al., 2010; Caldu et al., 2019; Andriushchenko et al., 2020).

Two aspects of the ability to recognize emotions can be distinguished: the accuracy of the differentiation between basic emotions and the sensitivity to their intensity. The main ability model of emotional intelligence by Mayer, Salovey and Caruso includes 4 groups of cognitive abilities: identifying emotions, using emotions, understanding emotions, and managing emotions (Mayer et al., 2004; Lyusin et al., 2016; Israelashvili et al., 2019; Pankratova, 2019).

Nowadays, many researchers also focus on the experimental study of brain mechanisms for emotion recognition and the presentation of emotional expressions, using methods for registering evoked brain potentials and other psychophysiological variables (Raz et al., 2013; Barbey et al., 2014; Lyusin et al., 2016; Liu et al., 2017; Barabanschikov et al., 2019; Alekseeva et al., 2019). As a practical implication, some studies demonstrate that emotional intelligence can be trained (enhanced) by psychological methods (Hodzic, et al., 2018). Like many other psychological traits, emotional intelligence is determined on one hand by genetic influences, and on the other by environmental influences. These include belonging to a particular ethnic culture, which regulates certain features of emotional expression. In addition, there are the effects of genotype-environment interaction and genotype-environment covariance on emotional expression (Pankratova et al., 2016; Dewi et al., 2018; Katrushova et al., 2019). Genes associated with emotional intelligence are involved in the serotonergic and dopaminergic neurotransmitter systems, and affect neuroplasticity (Kim et al., 2011; Takeuchi et al., 2015; Kosonogov et al., 2019; Ermakov et al., 2019; Brown et al., 2020). The catechol-o-methyltransferase (COMT) gene is associated with the activity of the striatum and prefrontal regions. This gene is simultaneously associated with aggression, motivation, and recognition of negative emotions. The brain-derived neurotrophic factor (BDNF) gene is associated with memory disorders, depression, moral emotions, features of perception of affective visual scenes, regulation of emotions, and stress resistance. The dopamine receptor D2 (DRD2) gene is associated with emotional stability, control over emotions and activation of limbic structures, as well as with successful emotion recognition. The serotonin receptor type II (HTR2A) gene is associated with depression, social introversion, and anxiety (Pecina et al., 2013; Gohier et al., 2014; Vrshek-Schallhorn et al., 2015; Alfimova et al., 2019; Lischke et al., 2019; Caldu et al., 2019; Vorobyeva et al., 2020; Redlich et al., 2020; Quan et al., 2021; Tian et al., 2021).

Association of genes and recognition of emotions, emotion expressions and emotion management are often considered by studies in which the participants are affected by disorders like schizophrenia and autism spectrum disorder, but not by the study of healthy individuals (Gadow et al., 2014; Liu et al., 2020; Qin et al., 2020; Halicka-Maslowska et al., 2021).

We hypothesized that emotional intelligence can be influenced by genetic factors. It can be associated with dominant or recessive alleles of the COMT, BDNF, DRD2 and HTR2A genes. The aim of this work is to compare emotional intelligence in mentally healthy carriers of the COMT, BDNF, DRD2 and HTR2A genotypes.

## Methods

### Participants

A sample of 280 volunteers (65.6% female; mean age = 19.7; SD = 2.9) participated in the study. The participants lived in the south of Russia: in Rostov-on-Don and Chaltyr (one of its rural localities), in Nalchik (Republic of Kabardino-Balkaria), and in Karachaevsk (Republic of Karachay-Cherkessia). All participants agreed to voluntarily participate in the study. They were all right-handed with normal (or corrected to normal) vision. All procedures were conducted in accordance with the declaration of Helsinki.

### Procedure

We used three methods to measure emotional intelligence: the Mayer-Salovey-Caruso Emotional Intelligence Test and two self-report questionnaires by Lyusin and Hall. The Russian version (Sergienko & Vetrova, 2010) of the Mayer-Salovey-Caruso Emotional Intelligence Test (*MSCEIT*; Mayer et al., 2003) consists of 141 items that reflect four branches: perceiving emotions, facilitating thought, understanding emotions, and managing emotions. All the items represent a task with multiple choice answers. Unlike with self-report questionnaires or cognitive evaluation, we applied the sample frequency procedure to analyse the responses. Thus, correct responses were not predetermined, but based on the frequency of each option in the studied sample. For each item, each option obtained a score correspondent to its frequency in the given sample of participants. In other words, if a participant selected the most frequent option in an item, they received a greater score of emotional intelligence for this item. For example, if a participant selected option 1 (“no disgust”) for item 4, and 60% of the whole sample also selected option 1, this participant would obtain a score of .60 for item 4. After that, the scores of all items were averaged in order to get the overall score of MSCEIT.

*EmIn* (Emotional intelligence) is a self-report measure by D. Lyusin (2006). It consists of 46 questions that describe five domains: understanding of one’s own and others’ emotions, management of one’s own and others’ emotions and control of expression. It showed a good reliability (.80), and confirmatory factor analysis revealed a 5-factor model, as proposed by the author. *EI* (Emotional intelligence) questionnaire by N. Hall (retrieved from Ilyin, 2001) is a wide-spread self-report measure of emotional intelligence. However, to our knowledge, it has not been subject to psychometric analysis. It consists of 30 questions with a 6-score scale.

DNA was extracted from buccal cells and the genotyping procedure was carried out using PCR (“Biological Solutions and Technologies”, Russia, Moscow). The analyzed DNA sections were the following: BDNF gene (rs6265, 68690G>A, Val66Met; genotypes: Val/Val, Val/Met, Met/Met), COMT gene (rs4680, 23753G>A, Val158Met; genotypes: Val/Val, Val/Met, Met/Met), HTR2A gene (rs6311 (Tr2), 4692G>A, genotypes: G/G, G/A, A/A; rs6313 (Tr3), 6230С>T, genotypes: C/C, C/T, T/T), DRD2 gene (rs1800497, 32806C>T; genotypes: : C/C, C/T, T/T).

As the data were distributed normally (all *p*s > .05, according to Kolmogorov-Smirnov tests), parametric analyses were applied. Correlations between emotional intelligence measures were calculated by the Pearson coefficient. Parametric analysis of variance with the Bonferroni correction for multiple comparisons was used to study the effects of different genotypes on the emotional intelligence scores. Partial eta-squared (η*_p_*^2^) was computed as a measure of effect size.

## Results

### Emotional intelligence measures

We found a positive correlation between the two self-report measures of emotional intelligence, EmIn by Lyusin and EI by Hall, r = .44, p < .001. Correlations between these self-report measures and MSCEIT were not significant (p > .05) (Table 1), despite research previously demonstrating a small correlation between MSCEIT and some self-report measures of emotional intelligence (Zeidner et al., 2005; Farrelly et al., 2007).

**Table 1:** Descriptive statistics and correlation coefficients between emotional intelligence measures

intelligence Emotional measures	Mean	*SD*	EmIn	EI
MSCEIT	.393	.044	.09	.08
EmIn by Lyusin	79.8	12.5		.44*
EI by Hall	25.1	27.4		

Note. *p<.05

### Association between СОМТ, BDNF, DRD2, HTR2A genotypes and emotional intelligence

**Figure 1.**
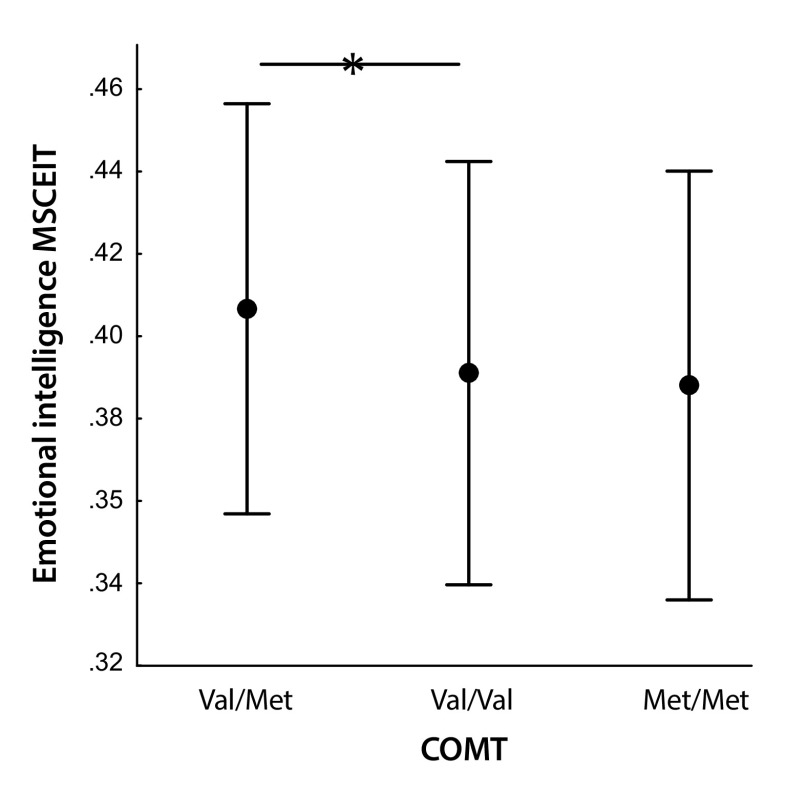
Emotional intelligence (MSCEIT) as according to the COMT genotypes Val/Met, Val/Val and Met/Met.

*Note. Points reflect means; whiskers reflect SDs. Horizontal lines mean that there are significant differences between indicated values.*

**Figure 2.**
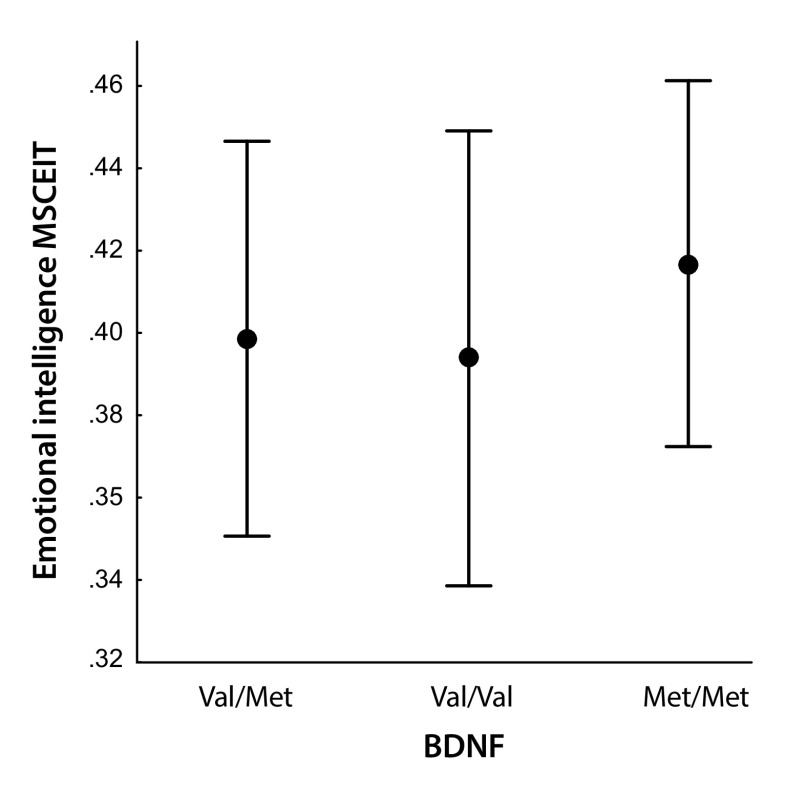
Emotional intelligence (MSCEIT) as according to the BDNF genotypes Val/Val, Val/Met and Met/Met.

*Note. Points reflect means; whiskers reflect SDs. Horizontal lines mean that there are significant differences between indicated values.*

We found that COMT genotypes had an effect on MSCEIT (*F* = 3.20, *p* = .042, η*_p_*^2^ = 0.02 (*Figure 1*). Post-hoc analysis showed that the carriers of the Val/Met genotype (*M* = .406, 95% *CI*: .397 – .415) had a higher level of emotional intelligence than carriers of the Val/Val genotype (*M* = .391, 95% *CI*: .381 – .401). The carriers of the Met/Met genotype did not statistically differ from other subsamples to any significant extent, although their emotional intelligence was the lowest (*M* = .387, 95% *CI*: .368 – .407). The study did not reveal BDNF genotypes to have an effect on MSCEIT scores (*p* > .05) (*Figure 2*).

**Figure 3.**
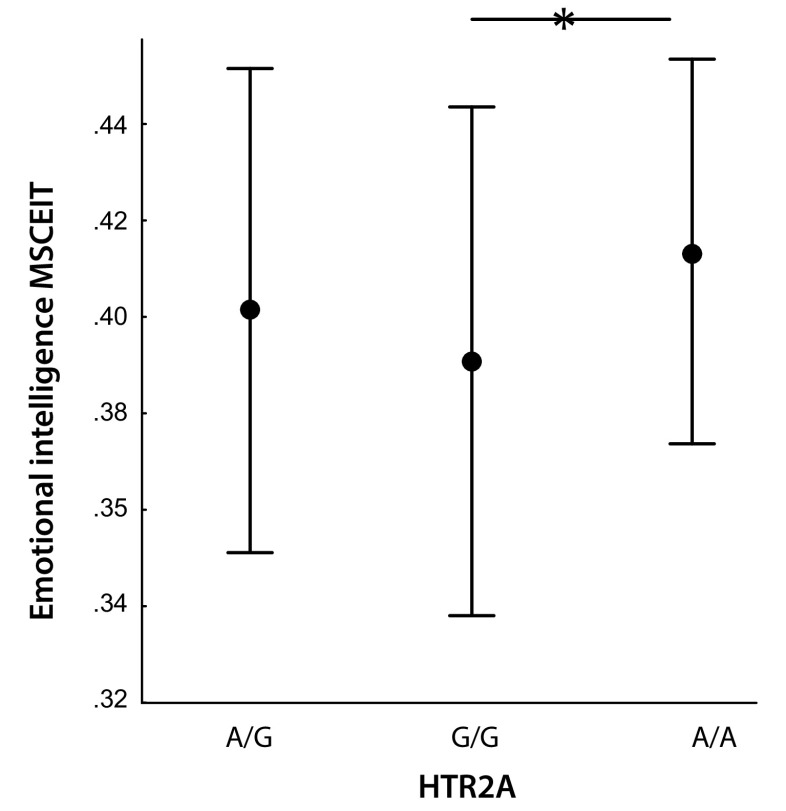
Emotional intelligence (MSCEIT) as according to the HTR2A genotypes A/G, G/G and A/A.

*Note. Points reflect means; whiskers reflect SDs. Horizontal lines mean that there are significant differences between indicated values.*

We found that differences in HTR2A genotypes did influence the MSCEIT score, *F* = 3.24, *p* = .041, η*_p_*^2^ = 0.03 (*Figure 3*). Post-hoc analysis showed that carriers of A/A (*M* = .414; 95% *CI*: .398 – .430) had a higher level of emotional intelligence than carriers of G/G (*M* = .391; 95% *CI*: .382 – .400). The A/G carriers did not differ from other subsamples in emotional intelligence (*M* = .401, 95% *CI*: .391 – .411).

**Figure 4.**
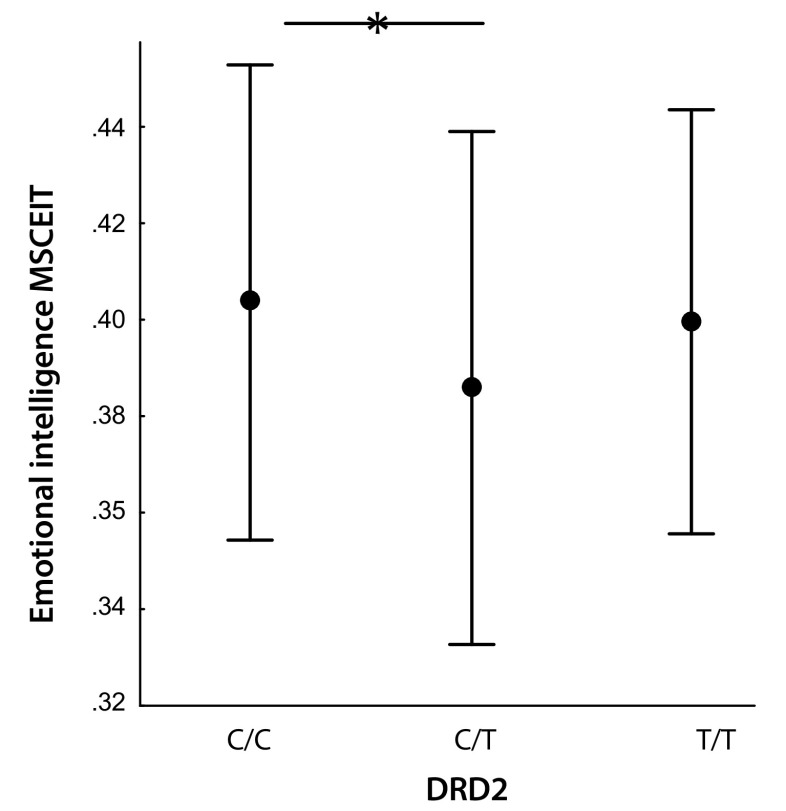
Emotional intelligence (MSCEIT) as according to the DRD2 genotypes C/C, C/T and T/T.

*Note. Points reflect means; whiskers reflect SDs. Horizontal lines mean that there are significant differences between indicated values.*

We also noted that different DRD2 genotypes affected the MSCEIT score, *F* = 3.42, *p* = .034, η*_p_*^2^ = 0.03 (*Figure 4*). Post-hoc analysis showed that C/C carriers (*M* = .405, 95% *CI*: .395 – .414) had a higher emotional intelligence than C/T carriers (*M* = .387; 95% *CI*: .377 – .397). Carriers of the T/T genotype did not differ from other subsamples in emotional intelligence (*M* = .401; 95% *CI*: .386 – .415).

As for the self-report measures of emotional intelligence, we did not find that differences in COMT, BDNF, HTR2A, DRD2 genotypes had any effect on EmIn or EI score (all ps > .05). Table 2 represents means and SDs of EmIn or EI scores in carriers of different COMT, BDNF, HTR2A, and DRD2 genotypes.

**Table 2:** EmIn or EI scores in carriers of different COMT, BDNF, HTR2A, and DRD2 genotypes.

		EmIn by Lusin		EI by Hall	
		mean	*SD*	mean	*SD*
COMT	Val/Val	80.7	14.4	26.3	28.4
	Val/Met	79.1	12.8	26.6	26.1
	Met/Met	80	10.5	27.9	31.6
BDNF	Val/Val	79.2	12.8	24.8	26.8
	Val/Met	81.2	16	26.8	28.1
	Met/Met	87.1	15.6	41.2	26.9
HTR2A	A/A	80.2	9.9	30.9	22.9
	A/G	80.2	14.9	26.9	28.3
	G/G	79.3	12.7	25.1	28
DRD2	C/C	80	13	24.8	27.8
	C/T	79.9	13.8	28.9	27.8
	C/C	80	13	24.8	27.8

## Discussion

In this paper we conducted a comparative study of emotional intelligence in carriers of various genotypes of the COMT, BDNF, HTR2A, and DRD2 genes. The objective method of measuring emotional intelligence (the Mayer-Salovey-Caruso test) showed much greater validity than self-reported (subjective) questionnaires, with regards to genetic differences.

### The catechol-o-methyltransferase (COMT) gene

We found that the Val/Met genotype (which determines the average duration of monoamine residence in the synaptic space) was associated with a higher level of emotional intelligence. In general, the heterozygous genotype of the COMT gene is associated with a higher adaptive potential and participants’ self-reports of effective emotion regulation in everyday life (Weiss et al., 2016; Bunyaeva et al., 2016), which is consistent with our data which saw a significantly higher level of emotional intelligence in representatives of this group. The catechol-o-methyltransferase (COMT) gene influences changes in amygdala activity and amygdala-prefrontal connectivity during face processing (Lonsdorf et al., 2011; Surguladze, S. A. et al., 2012). It has been shown that insufficient or excessive catecholaminergic activity associated with the COMT genotype is undesirable for the ability to maintain stable focus of attention during visual perception (Shalev et al., 2019), which can be important for visual recognition of emotions.

### The brain-derived neurotrophic factor (BDNF) gene

It is known that the presence of the Met allele of the BDNF gene is associated with reduced brain-derived neurotrophic factor secretion, which is associated with the transition from plasticity to stability in neural networks (Egan et al., 2003; Brown et al., 2020). This allows us to assume the possible existence of links between the level of emotional intelligence and the presence or absence of the Met allele. However, in our work we found that the genotypes of the BDNF gene are not associated with the level of emotional intelligence.

### The dopamine receptor D2 (DRD2) gene

In this work, it was found that the presence of the major C/C genotype (with a high number of dopamine D2 receptors on the presynaptic membrane) is associated with higher levels of emotional intelligence according to the Mayer-Salovey-Caruso test. At the same time, the heterozygous C/T genotype (with an average number of dopamine D2 receptors on the presynaptic membrane) is associated with a lower level of emotional intelligence. Consequently, a high number of dopamine D2 receptors on the presynaptic membrane is associated with higher levels of emotional intelligence. The dopamine receptor D2 (DRD2) gene is a part of the dopamine neurotransmitter system. The presence of at least one minor allele in the genotype leads to a decrease in the number of dopamine D2 receptors on the presynaptic membrane and increases a person’s propensity to develop various addictions (Kibitov, 2011). It was shown that the presence of minor DRD2 alleles in both healthy subjects and patients with schizophrenia was associated with worse emotion recognition (Alfimova et al., 2019).

### The 5-hydroxytryptamine receptor 2A (HTR2A) gene

We found that the presence of a minor A/A genotype was associated with a high level of emotional intelligence. The major G/G genotype was associated with a lower level of emotional intelligence. Our results do not contradict the results of another study, where it was indicated that the G allele of rs6311 was related to higher scores on the Toronto Alexithymia Scale compared to the AA genotype (Li et al., 2020). In studies on samples of patients with schizophrenia, it was shown that schizophrenia patients more often carry the major genotype C/C of the HTR2A gene to a statistically significant extent (this corresponds to G/G, since the loci rs6313 C>T and rs6311 G>A are in non-equilibrium coupling), while having a low density of serotonin 2A receptors in the brain (Polesskaya et al., 2002; Parsons et al., 2004). In a recent study performed on healthy subjects using positron emission tomography, there were no statistically significant differences in the density of serotonin 2A receptors between carriers of different genotypes of the HTR2A gene, but the trend (without statistical significance) was the same as in the samples of patients (Spies et al., 2020). In the same vein, we can assume that the association of the minor A/A genotype and a high level of emotional intelligence may be accompanied by a tendency to a higher number of serotonin 2A receptors on the presynaptic membrane and may be affected by epigenetic mechanisms. For example, studies of the epigenetic mechanisms of depression, such as DNA methylation, as well as genotype-environment interaction, have shown that depression is associated with the exposure to various environmental risk factors, such as stress, childhood abuse, and stressful life events (Lin, E. et al., 2019). In a study by Brown and colleagues, devoted to the analysis of the epigenetics of emotion regulation disorders, it was shown that the most important role in these processes is that of methylation, which regulates the activity of genes without changing the DNA sequence. The important role of genes in the implementation of the serotonin, dopamine and noradrenaline systems, as well as the limbic-hypothalamic axis of the adrenal glands (l-HPA) is also noted (Brown et al., 2020).

## Conclusion

On the basis of the conducted research, some generalizations can be formulated. This work was one of the first studies to investigate the influence of genetic factors on emotional intelligence in healthy respondents. The carriers of the Val/Met genotype of the COMT gene, A/A genotype of the HTR2A gene and C/C genotype of the DRD2 gene showed the highest levels of emotional intelligence. These differences, according to our data, may also be caused by genetic differences in serotonin and dopamine neurotransmitter systems, which control the duration of both serotonin and dopamine in the intersynaptic space. These include the 5-hydroxytryptamine 2A receptor (HTR2A) gene, the dopamine receptor D2 (DRD2) gene, and the catechol-o-methyltransferase (COMT) gene.

The results obtained can form the basis for further research, possibly involving more participants. Recently, a neural model of emotional intelligence was proposed, which offered plausible targets for improving emotional intelligence with training (Smith et al., 2018). Taking into account genetic characteristics will make such training more individualized.

Emotional intelligence research is currently one of the most dynamically developing areas of modern psychological science, and this trend only intensifies with the increase in the number of risks faced by a modern person (e.g., restrictions due to a pandemic). The level of emotional intelligence in an individual can be regarded as the single most important personal psychological resource that can ensure successful adaptation in society, since it allows its owner to recognize and express, regulate, and manage both his own emotional manifestations and the emotions of other people. In this work, based on a sample of young people in the South of Russia living in the Rostov region and the republics of Kabardino-Balkaria and Karachay-Cherkessia, a comparative study of the diagnosis of emotional intelligence using three different methods was carried out. These methods were the self-report questionnaires of Hall and Lyusin and the objective methodology of Mayer-Salovey-Caruso.

The manifestations of emotional intelligence can have their own characteristics, caused by both genetic factors (depending on the frequency of occurrence of homozygous dominant and recessive or heterozygous genotypes) and environmental influences (training and education in ways of manifesting emotions). The study of emotional intelligence acquires particular relevance if and when it is based on the use of natural scientific methods that allow a deeper understanding of the nature of the studied psychological phenomena.

## Limitations

Finally, we admit that our work has several limitations. Thus, we have not equalized the gender and ages of our subsamples. Future studies could collect data for more genes, such as the oxytocin receptor (OXTR), which is associated with empathy traits (Luo et al., 2015), or the dopamine D4 receptor (DRD4) related to social behavior (Uzefovsky et al., 2014) and others.. We also suggest that larger samples could be examined in order to expand upon the knowledge of the association between genetics and personality.
